# Transcriptional regulation in skeletal muscle and adipose tissue of lean and obese colony cats

**DOI:** 10.1371/journal.pone.0331028

**Published:** 2026-03-27

**Authors:** Rahel Rigotti, Annette Liesegang, Holly Dawson, Daniel Brugger, Diana Frey, Georgina Samaha, Bianca Haase, Brigitta Wichert

**Affiliations:** 1 Institute of Animal Nutrition and Dietetics, Vetsuisse Faculty, University of Zurich, Zurich, Switzerland; 2 Sydney School of Veterinary Science, Faculty of Science, The University of Sydney, Sydney, New South Wales, Australia; 3 Department of Rheumatology, University Hospital Zurich, Zurich, Switzerland; 4 Sydney Informatics Hub, Core Research Facility, The University of Sydney, Sydney, New South Wales, Australia; University of Life Sciences in Lublin, POLAND

## Abstract

This study analysed gene expression profiles in white adipose and skeletal muscle tissues of cats genetically predisposed to obesity and wildtype cats to uncover molecular mechanisms regulating obesity. It also examined how these profiles altered after change in body fat through changes in the amount of food. For this purpose, 3 female cats with phenotype lean (GL) and 3 female cats with phenotype obese (GO) were included in the study. Fat and muscle samples were obtained twice from these cats (T1 = all cats lean, T2 = after ad libitum feeding GL still lean, GO obese). In addition Body Conditions Score (BCS) was measured at both time points, body fat content was determined using DEXA and blood samples were taken to measure glucose levels. BCS revealed a significant positive linear regression with body fat content. Comparing BCS and blood glucose level, no significant difference was observed. The study found distinct gene expression changes in muscle and adipose tissues when comparing obese and lean cats at two time points (T1 and T2). In muscle tissue, group differences between obese and lean cats were detected in three downregulated and four upregulated genes when all cats were lean, while only two genes were upregulated when one group of cats became obese (T2). For adipose tissue, fewer differences were observed, with minimal changes in gene expression between the two groups, especially at T2, where lean cats showed only one downregulated gene and obese cats showed no change. The regulated genes identified are mainly involved in tissue maintenance and cellular turnover, either specific to the tissue studied or with broader roles. No clear phenotypic trend in gene expression was observed. Differences emerged mainly within the obese group over time, reflecting adaptive feeding responses, while tissue-specific differences in the transcriptome were most prominent. Overall, phenotype had no distinct effect on gene expression patterns.

## Introduction

Body weight maintenance is regulated by various mechanisms that influence energy intake and expenditure. Many cats are overweight or even obese. A study of over 9000 feline medical records in California over 9 years showed a prevalence of 41% [1]. Another study in the USA, which also analyzed the medical records of over 1.3 million cats, showed a prevalence of up to 47% in adult cats [2]. Obesity in cats is influenced by many risk factors such as neutering, sex, age, housing, as well as the owner's attitude towards obesity [1,3,4]. Due to the increasing prevalence of obesity and diabetes mellitus in men and companion animals, earlier studies have investigated the underlying physiological and molecular mechanisms of weight gain across species [5–9]. In humans, obesity is associated with chronic low-grade inflammation of adipose tissue, characterized by the infiltration of macrophages and the release of inflammatory cytokines [10–13]. In contrast, studies have shown that anti-inflammatory pathways are upregulated after extensive weight loss, leading to a decline in tissue and systemic inflammation [14]. A study using CT scans in cats with varying body condition scores (BCS) revealed that visceral fat triggers inflammatory responses, leading to low adiponectin and high Serum Amyloid A (SAA) levels [15]. Experimental data from cats indicate similar interactions, with RNAseq studies confirming the upregulation of pro-inflammatory genes in adipose tissue of obese individuals [16]. No detectable changes in inflammatory cytokine mRNA have been identified in obese cats [17]. This suggests that the development of obesity and insulin resistance in cats may be influenced by factors other than tissue-related inflammation.

Transcriptomic analyses in various species, including pigs, cows, mice, chicken and sheep, have identified differentially expressed genes associated with obesity-related pathways such as lipid metabolism, immune responses, and protein binding [18–24]. Gene expression analyses in subcutaneous white adipose tissue samples from children reveal significant alterations in gene expression correlated with increasing body mass index standard deviation scores, with a notable proportion of transcripts showing both upregulation and downregulation [25]. These studies highlight the complexity of obesity-related gene expression changes and the involvement of multiple biological processes across different animal models.

Skeletal muscle tissue plays a crucial role in whole-body energy homeostasis, glucose distribution, and plasma glucose regulation [26]. Studies in various animal models, including pigs, dogs, monkeys, and cats, have identified gene expression changes in muscle tissue associated with lipid metabolism, proteolysis, glycogen synthesis, and insulin signalling [17,27–30].

The present study investigated the global transcriptome profile of white adipose and skeletal muscle tissue of queens, comparing cats genetically predisposed to obesity with wildtype cats to identify molecular mechanisms involved in the regulation of obesity. Furthermore, we investigated changes to global transcriptome profiles following the reduction of body fat content by means of dietary restriction. Our hypotheses were, that (A) the body fat content is associated with the transcriptome profile in white adipose and skeletal muscle tissue; and (B) a reduction of the body fat content changes these transcriptome profiles with respect to the type of regulated pathways and the direction of regulation. This study has the potential to guide future targeted research on feline obesity.

## Materials and methods

### Animals

Housing of cats complied with Swiss Animal Welfare legislation and experiments were approved by the Swiss veterinary authorities (ZH128/2014), and cats were housed in groups of two to four. A total of six female European Shorthair cats from an experimental cat colony described previously [7] were included in this study. All cats were fed a commercial diet (Swiss Professional Kitten Chicken, Biomill SA, Herzogenbuchsee, Switzerland) ad libitum and based on their body condition score at the age of eight months (9 point scale BCS system; according to LaFlamme et al. [31], three lean females (Group GL, BCS ≤ 5.5/9) and three overweight females (Group GO, BCS ≥ 6/9) were selected. For each cat, the BCS was determined independently by the same two people. The three cats classified as overweight underwent a weight-loss diet, to ensure, all six cats were lean (BCS ≤ 5.5/9) at the beginning of the experiment. At this timepoint the three cats from GL were 2.25 years, 1.83 years and 0.75 years old and weighed 4.05 kg, 3.1 kg and 2.95 kg. In GO one cat was 1.17 year old, the other two were 4.59 years old and weighed 3.65 kg, 3.05 kg and 4.25 kg respectively. Thereafter, all six cats were fed ad libitum until the three GO cats achieved a BCS ≥ 6/9. All animals were intact and clinically healthy. No cat was euthanized for the research. In order to minimize the invasiveness of the examinations, the animals were trained for fear-free handling and solid pain medication was administered if necessary.

### Blood samples and body fat content

**Fig 1:**
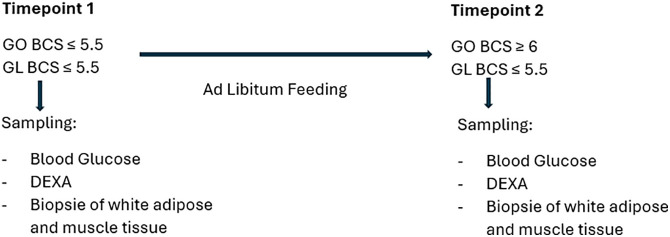
Timeline of the study. Study begins at Timepoint 1 with all the cats from group obese (GO) and group lean (GL) with a BCS of ≤ 5.5. Blood glucose measurement and dual X-ray absorptiometry as well as biopsy samples of white adipose and muscle tissue were performed. All cats were fed ad libitum from then on until GO had a BCS of ≥ 6, while GL maintained a BCS of ≤ 5.5. Measurement of blood glucose and dual X-ray absorptiometry as well as biopsies of white adipose and muscle tissue were performed again.

From each cat a blood sample was taken from the vena cephalica using a 20 gauge needle to determine the blood glucose level in a fasting state using a glucometer (AlphaTrak® 2, Zoetis Inc., Kalamazoo, MI 49007) immediately prior to sedation for the Dual-X-ray-Absorptiometry (DEXA) according to Ghielmetti [32] to determine body fat content. For this procedure, cats were sedated with 0.2 mg/kg Butorphanol i.m. (Morphasol^**®**^-4 ad us. vet., solution for injection) and 0.05–0.075 mg/kg Medetomidin i.m. (Dorbene^**®**^ ad us. vet., solution for injection or Domitor^**®**^ ad us. vet., solution for injection). Each cat was measured at two different timepoints, defined by their BCS. The cats from GO had a BCS of ≤ 5.5 at Timepoint 1 (T1) and a BCS ≥ 6 at Timepoint 2 (T2). The cats from GL had always a BCS < 5.5 (T1 and T2) and where measured in each case parallel to the measurements of the cats from GO. An accurate timeline is shown in Fig 1.

### Tissue samples

Biopsies (100−150 mg) of subcutaneous adipose tissue (inguinal) and skeletal muscle tissue (Musculus tibialis cranialis) were taken under general anaesthesia at T1 and T2. After fasting for approximately 17 hours, cats were anesthesized with 0.4 mg/kg Butorphanol i.m. (Morphasol^**®**^-4 ad us. vet., solution for injection) and 0.04 mg/kg Medetomidin i.m. (Dorbene^**®**^ ad us. vet., solution for injection or Domitor^**®**^ ad us. vet., solution for injection) as a praemedication and 4 mg/kg Ketamine i.m. (Ketanarkon^**®**^ 100 ad. us. vet., solution for injection or Narketan^**®**^ 10 ad. us. vet., solution for injection). To prevent post-operative pain and inflammation, cats received 0.3 mg/kg Meloxicam s.c. (Metacam^**®**^ 5 mg/ml ad us. vet., solution for injection) preoperative. Tissue samples were immediately placed in RNAlater^**®**^ solution (Sigma-Aldrich, St. Louis, MO, USA) and stored at −80°C.

### RNAseq

Total RNA was extracted from all tissue samples using the Trizol protocol (Invitrogen, Carlsbad, CA, USA) and quality and quantity was tested using the NanoDrop^TM^ Spectrophotometer (Thermo Fisher Scientific, Waltham, MA, USA) and the BioAnalyzer (Agilent Technologies, Inc., Santa Clara, CA, USA). Samples that passed the quality check (amount: 400ng total RNA; concentration: 20–50ng/ul; minimum volume: 20ul) were sent to an external Service provider (Ramaciotti Centre for Genomics, University of New South Wales) for individual library preparation using the TruSeq RNA Sample Preparation Kit (Illumina, San Diego, CA, USA). Individual libraries were pooled, and next-generation sequences generated using one NovaSeq S1 flow cell (Illumina, San Diego, CA, USA).

### Statistical and RNAseq data analysis

Data analysis of BCS, body fat and blood glucose were performed with SAS Studio 3.81 (SAS Institute Inc., Cary, North Carolina, USA). The relationship between BCS and body fat and blood glucose, respectively was analysed by linear regression (y = a + bx) with the procedure REG and BCS as the independent variable.

RNA-seq data analysis was conducted using R (Version 4.0.3). The *Felis catus* 9.0 reference genome and annotation files (release 102) were downloaded from Ensembl. Raw read quality was assessed using FastQC (Version 0.11.9), and adaptor contamination was checked with Fastp. FastqScreen (Version 0.14.1), in combination with Bowtie2 (Version 2.4.1), was used to detect potential contamination from non-target species. On the other hand, STAR (Version 2.7.7a) was used for exploratory mapping-based QC to assess general alignment quality. Transcript quantification was performed using Kallisto, a pseudoalignment-based tool. Gene-level count data generated by Kallisto were analyzed with edgeR (Version 3.32.0). Count normalization was performed using the trimmed mean of M-values (TMM) method to account for differences in library size. Generalized linear models (GLMs) were applied to model the effects of different experimental conditions, and the likelihood ratio test (LRT) was used to identify statistically significant differentially expressed genes. Annotation of significantly expressed genes within each of the tissues was performed using the OMIM database (https://www.omim.org/) and ensembl (https://www.ensembl.org/). We looked for differentially expressed genes (DEG) which were defined by a false discovery rate (FDR) of <0.2 and a log fold change (logFC) of >1.

## Results

### BCS – Body fat content

**Fig 2:**
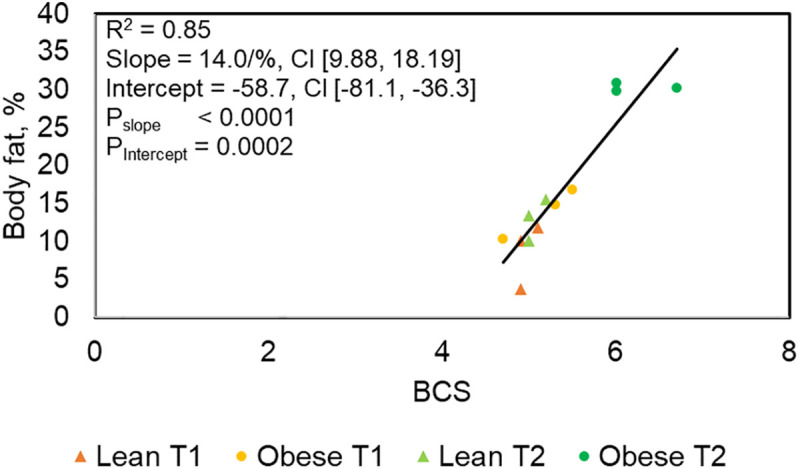
Linear regression analysis of BCS against the body fat content. A total of six cats (3 lean, 3 obese) were measured for BCS and body fat content (%) over two timepoints, T1 = before ad libitum feeding; T2 = after ad libitum feeding. n = 12 datapoints. Body condition scoring according to LaFlamme.

In the lean condition (BCS ≤ 5.5/9) the mean value of the fat content over all 6 cats was 12% (standard deviation (SD) ± 4.00). In the overweight condition (BCS ≥ 6) the mean value over the three cats from GO was 27% (SD ± 5.32). Overall BCS revealed a significant positive linear regression with body fat, with an increase of 14 percentage points per unit increase in BCS (P < 0.0001) (Fig 2).

### BCS – Blood glucose

In the lean condition (BCS ≤ 5/9) the mean concentration of blood glucose was 4.7 mmol/l (SD ± 0.51). In the obese condition (BCS ≥ 6) the concentration was 4.6 mmol/l (SD ± 0.26). No significant interaction was observed between both measures based on linear regression analysis at P = 0.68 (data not further shown).

### Differential expression analysis

The pooled libraries resulted in a total of 1.6B reads. The 12 muscle tissue samples obtained an average of 67,287,181 raw reads per sample, whereas the 12 adipose tissue samples obtained an average of 81,341,386 raw reads per sample. Quality assessment revealed no adapter contamination and high sequence quality (phred>30) and all reads remained for further analyses. For the muscle tissue on average 61.5% of the reads mapped to the feline reference sequence and for the adipose tissue it was 25.9%. The DESeq2 analysis identified 17,683 significantly differentially expressed genes (DEGs) (adjusted P-value < 0.05) between adipose and skeletal muscle tissue. Of these, 11,575 genes were upregulated in adipose tissue (log2FC > 1), while 6,108 were upregulated in skeletal muscle (log2FC < −1).

**Fig 3:**
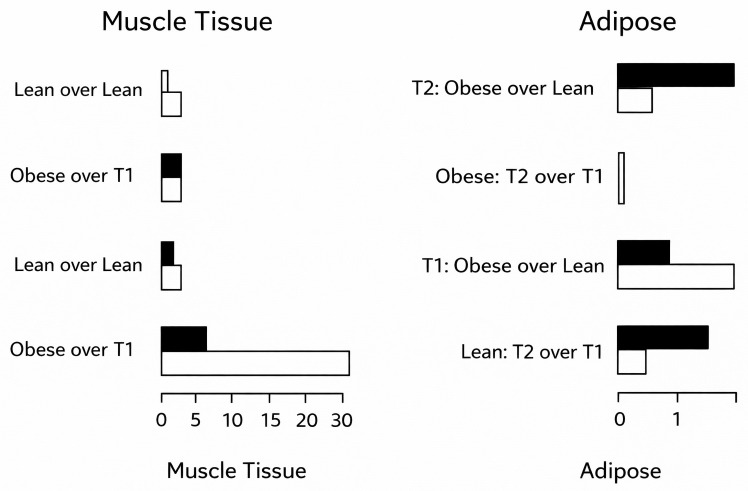
Number of differentially expressed genes in muscle tissue (left) and adipose tissue (right). n = 6 cats (3 cats phenotype lean, 3 cats phenotype obese). Timepoint 1 (T1), all 6 cats lean due to preliminary energy restricted feeding of the 3 phenotypically obese cats until they reached a BCS of 5.5. Timepoint 2 (T2), cats segregated again into 3 lean and 3 obese individuals, respectively, due to ad libitum feeding. white = upregulated genes of phenotype obese compared to phenotype lean respectively within phenotype itself T2 compared to T1, black = downregulated genes of phenotype obese compared to phenotype lean respectively within phenotype itself T2 compared to T1. Unit = exact number of differentially expressed genes. Differential gene expression analysis based on RNA sequencing, assuming FDR < 0.2 and logFC > 1; FDR = false discovery rate; logFC = Log fold change.

**Fig 4:**
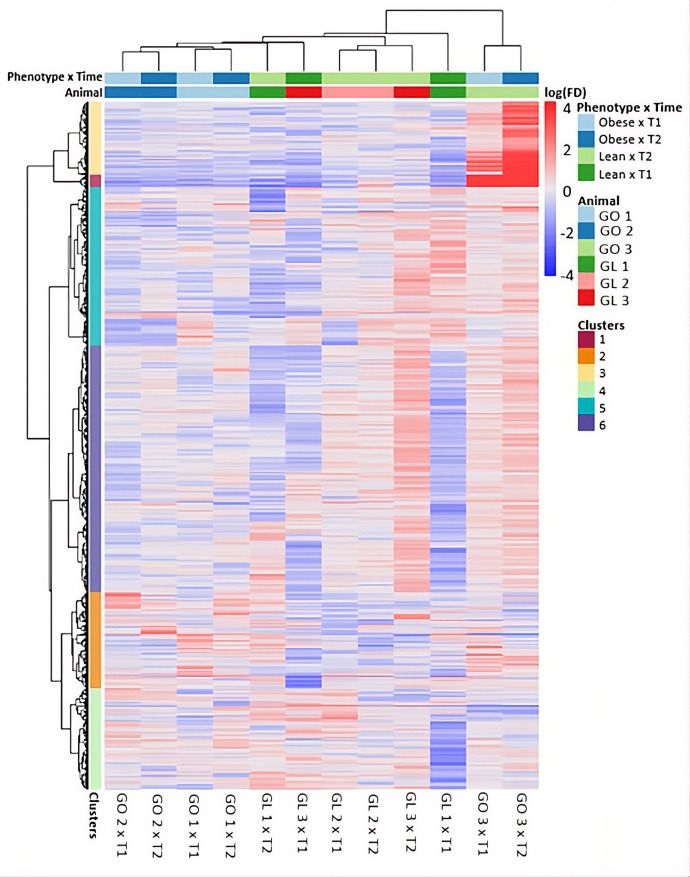
Cluster Heat Map of adipose tissue samples from cats sampled at two different time points and expressing varying genetic predisposition to obesity. n = 6 cats (3 cats without genetic predisposition to obesity (GL 1, GL 2, GL 3), 3 cats with genetic predisposition to obesity (GO 1, GO 2, GO 3; all cats belonged to European Shorthair cats from an experimental cat colony. T1 = Timepoint 1 (all cats lean (BCS ≤ 5.5) after energy restricted feeding prior to sampling), T2 = Timepoint 2 (Cats segregated into three lean (BCS ≤ 5.5) and three obese cats (BCS ≥ 6) after ad libitum feeding from T1 on. Genetic predisposition of half of the study cats was induced by crossbreeding.

**Fig 5:**
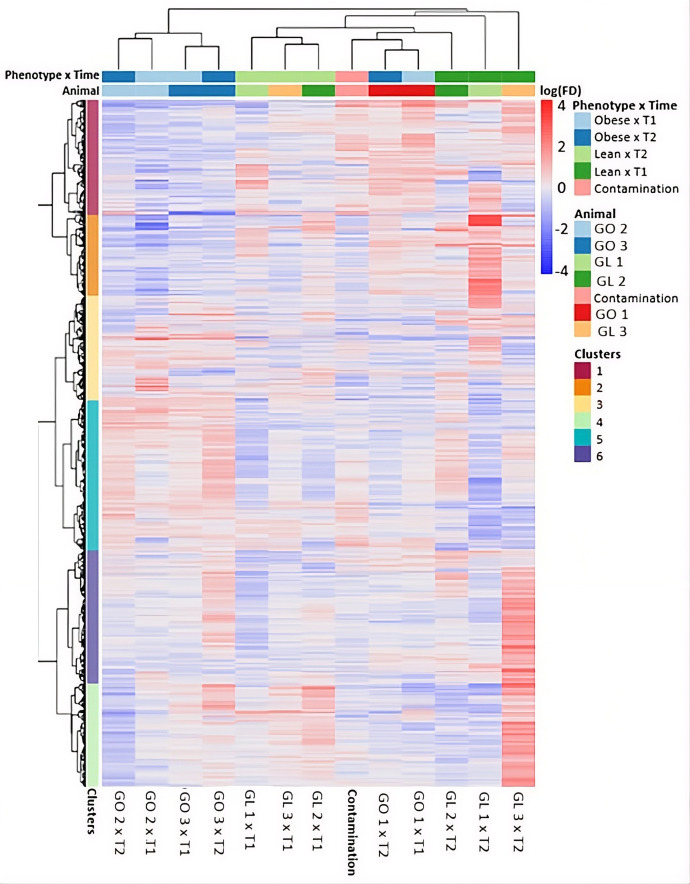
Cluster Heat Map of muscle tissue samples from cats sampled at two different time points and expressing varying genetic predisposition to obesity. n = 6 cats (3 cats without genetic predisposition to obesity (leanGL1, GL2, GL3), 3 cats with genetic predisposition to obesity (obese, GO1, GO2, GO3); all cats belonged to European Shorthair cats from an experimental cat colony. T1 = Timepoint 1 (all cats lean (BCS ≤ 5.5) after energy restricted feeding; T2 = Timepoint 2 (Cats segregated into three lean (BCS ≤ 5.5) and three obese cats (BCS ≥ 6) after ad libitum feeding from T1 on. Genetic predisposition of half of the study cats was induced by crossbreeding. “Contamination” represents an unexpected subcluster which, according to annotation, represents a contamination of adipose tissue with non-adipose tissue. Based on the expression pattern it was concluded the non-adipose tissue to be muscle tissue from T1.

A total of 77 DEGs were identified in adipose tissue using the initial sampling timepoint (BCS ≤ 5.5/9), with 60 genes upregulated and 17 downregulated in obese cats. At the second timepoint (BCS ≥ 6/9), only five genes were differentially expressed, including one upregulated and four downregulated in obese cats. In skeletal muscle, six DEGs were identified at BCS ≤ 5.5/9 (four upregulated and two downregulated), and 25 DEGs at BCS ≥ 6/9 (14 upregulated and 11 downregulated in obese cats). These results are visualized in Fig. 3. When muscle gene expression of obese and lean cats at T1 were compared, three genes were downregulated and four upregulated in obese cats. At T2, two genes were upregulated. Within-group longitudinal comparisons (T2 vs. T1) revealed four upregulated and two downregulated genes in the obese group, and 29 upregulated and six downregulated genes in the lean group. In adipose tissue, one gene was upregulated and four downregulated at T1, and two genes were downregulated at T2 between phenotypes. Within-group comparison for adipose tissue showed no DEGs in obese cats and one downregulated gene in lean cats. These expression profiles between phenotypes were confirmed by cluster analysis (Figs 4 and 5).

S1 and S2 Tables show the annotation of the aforementioned differently regulated transcripts from muscle and white adipose tissue, respectively. Looking at the set of regulated genes and their functions reveals predominantly such that are involved in the regulation of tissue maintenance and cellular turnover either specific for the respective tissue under study or in a more general manner – like

*LDL receptor related protein 5* and *frizzled related protein* or *monoacylglycerol O-acyltransferase 3*.

In addition, gene set enrichment analysis was carried out for both adipose and muscle tissue. The results are shown in S1 to S8 Figs.

Figs 4 and 5 represent heat maps of regulated genes. No clear trend of phenotype could be seen. There is uniformity between animals, reflecting the high degree of relatedness of the individuals. Differences are only seen within the obese animals over time, reflecting the adaptive feeding during the experiment. Otherwise, tissue differences in the transcriptome are most apparent. No clear effect of the phenotype on the expression pattern of the genes could be determined.

**Fig 6:**
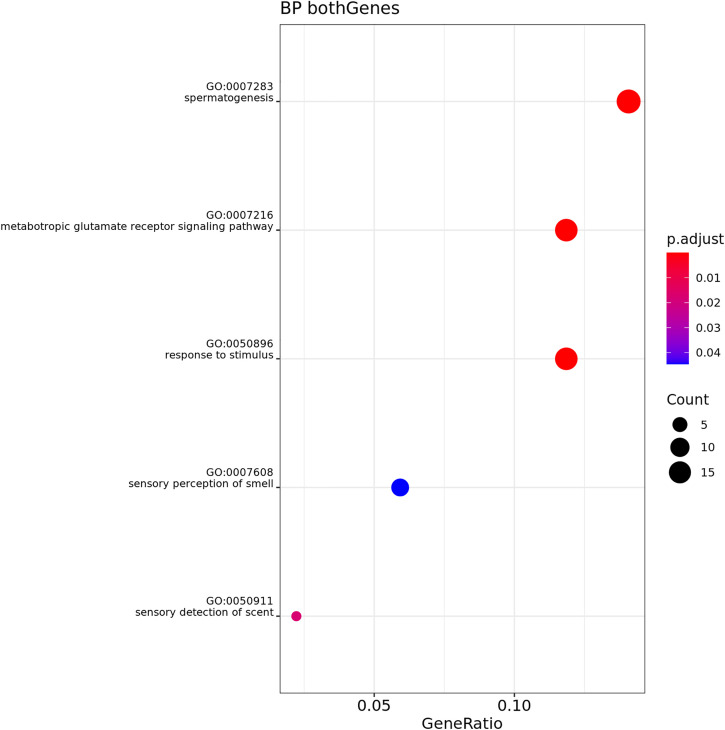
Gene Ontology Biological Process enrichment analysis (ORA) of differentially expressed genes in adipose tissue, n = 3 cats phenotype lean, comparing Timepoint 1 (T1) and Timepoint 2 (T2). The dot plot displays the top significantly enriched terms. The x-axis shows the gene ratio (number of DE genes in a term divided by total number of genes in that term). Dot size corresponds to the count of DE genes per term, and color represents adjusted p-values (red = more significant).

**Fig 7:**
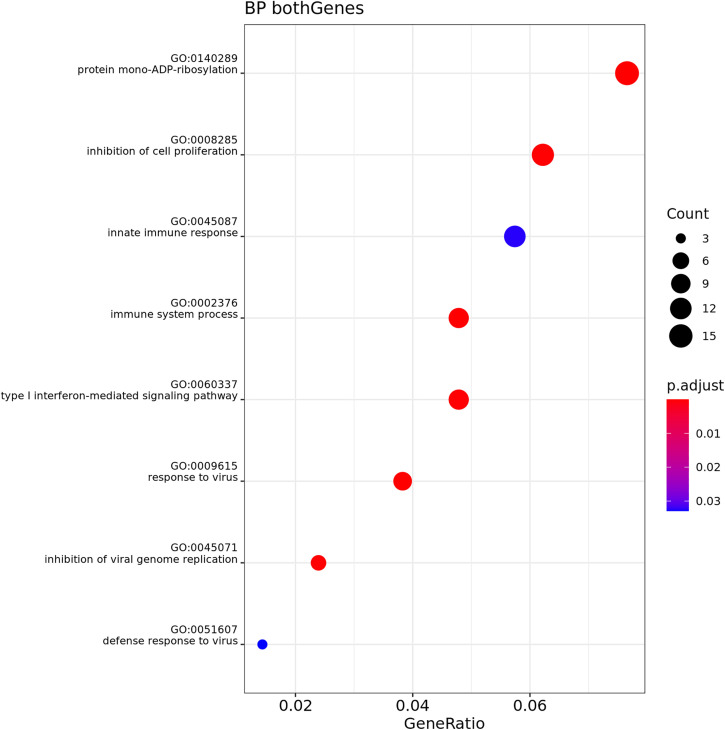
Gene Ontology Biological Process enrichment analysis (ORA) of differentially expressed genes in adipose tissue, n = 6 cats (3 cats phenotype lean (GL), 3 cats phenotype obese (GO)), comparing GO over GL at Timepoint 1 (T1). The dot plot displays the top significantly enriched terms. The x-axis shows the gene ratio (number of DE genes in a term divided by total number of genes in that term). Dot size corresponds to the count of DE genes per term, and color represents adjusted p-values (red = more significant).

**Fig 8:**
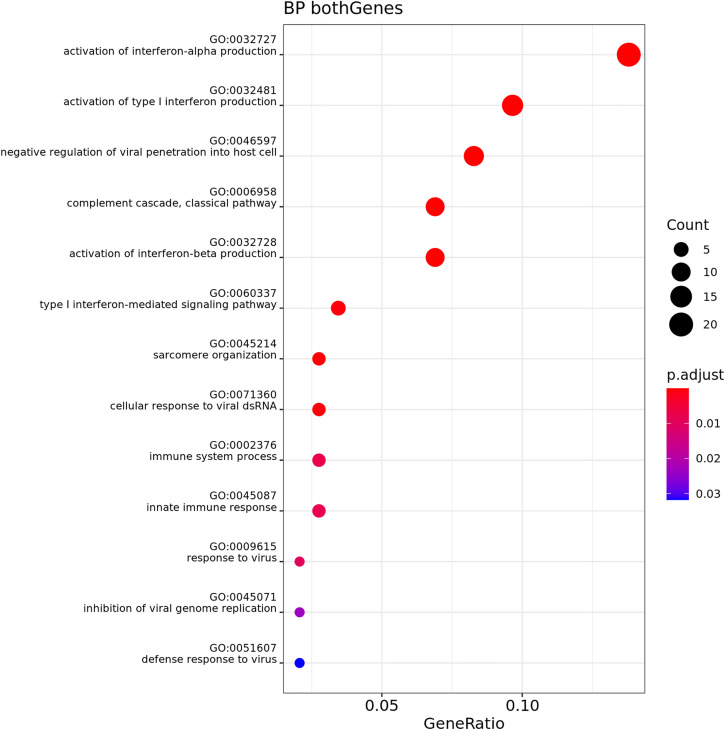
Gene Ontology Biological Process enrichment analysis (ORA) of differentially expressed genes in adipose tissue, n = 3 cats phenotype obese, comparing Timepoint 1 (T1) and Timepoint 2 (T2). The dot plot displays the top significantly enriched terms. The x-axis shows the gene ratio (number of DE genes in a term divided by total number of genes in that term). Dot size corresponds to the count of DE genes per term, and color represents adjusted p-values (red = more significant).

**Fig 9:**
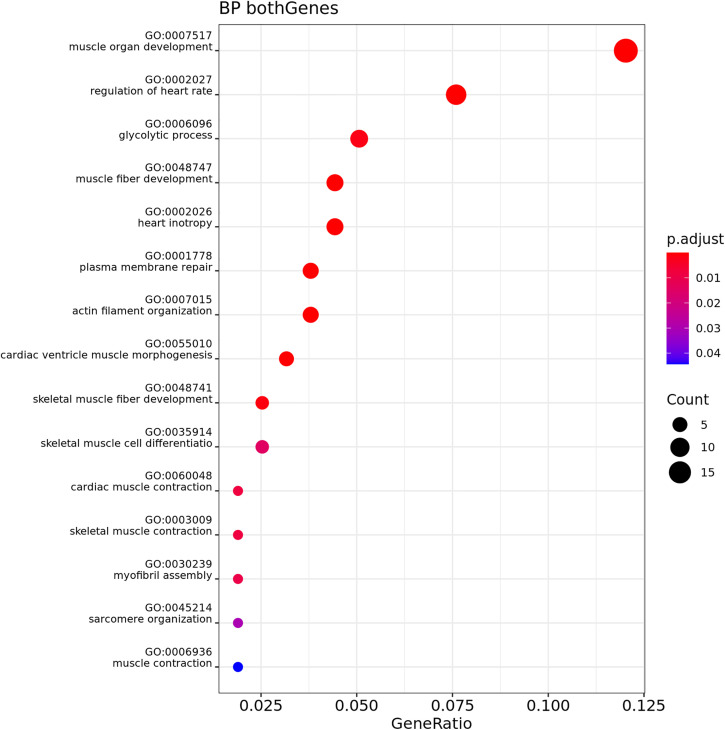
Gene Ontology Biological Process enrichment analysis (ORA) of differentially expressed genes in adipose tissue, n = 6 cats (3 cats phenotype lean (GL), 3 cats phenotype obese (GO)), comparing GO over GL at Timepoint 2 (T2). The dot plot displays the top significantly enriched terms. The x-axis shows the gene ratio (number of DE genes in a term divided by total number of genes in that term). Dot size corresponds to the count of DE genes per term, and color represents adjusted p-values (red = more significant).

Over-Representation Analysis (ORA) was performed for both muscle and adipose tissue, comparing GL and GO at T1 and T2. Muscle tissue showed no significant results. Figs 6–9 show the results for biological process (BP) in adipose tissue.

## Discussion

### Physiological measures

The cats used in this study showed a segregating overweight phenotype in a previous study, suggesting that genetic factors most likely determine the prevalence to their phenotypes [7]. This was confirmed by the study of Wichert et al. [33] using cats of the same cat family to reinduce obesity by *ad libitum* feeding of standard feed after a dietary weight loss program. A recent study by Opsomer et al. [34] using data from the same colony of cats showed a positive correlation between maternal phenotype (lean or obese) and the likelihood of kittens to develop obesity. The fact that we did not find pronounced differences between the transcriptome of phenotypic lean and obese cats at given timepoints, suggests that the regulation of feline weight development does not occur at the transcriptional rather than the post-transcriptional level, which may have involved an epigenetic component given the results of Opsomer et al. [34]. The latter has yet to be confirmed in follow-up studies.

The BCS is a subjective semi-quantitative method for the evaluation of body condition [31]. If appropriately conducted by the same two persons, it produces reproducible results as demonstrated on various occasions. Consequently, we observed the expected correlation between BCS and body fat as has been described earlier [35,36]. The reliability of the feline BCS system appears to be true also under conditions of minor differences in body fat, since the present study observed animals that even in the most “extreme” cases were still below the cut-off (BCS 7), which is considered the threshold from minor to heavy obesity. Therefore, overall BCS is a useful tool to determine body composition of cats non-invasively and quickly. Nevertheless, for scientific use it seems necessary to confirm this with help of a reliable method like DEXA.

In the present study, blood glucose concentration was within the range for healthy cats (4 mmol/l – 10 mmol/l) [37] in all animals irrespective of genetic precondition or time of observation. This demonstrates that the cats at the onset of obesity were still able to regulate their blood glucose, hence conditions could still be interpreted as physiological. However, it must be highlighted that an earlier study conducted with the same experimental cat family saw a positive correlation between BCS and insulin sensitivity as assessed by a glucose tolerance test, which is supposed to be more sensitive in the detection of physiological dysregulation than blood glucose alone [38]. It is therefore not clear, whether the cats of the present study already showed early signs of insulin resistance. However, earlier data obtained in dogs suggest that overweight animals (BCS 6) did not differ from lean dogs (BCS 4–5) in terms of insulin sensitivity or inflammatory status. Only if BCS rose above 7 (heavy obesity), the dogs showed physiological dysregulation [39]. In conclusion, slightly overweight cats are presumably still able to regulate their glucose metabolism within ranges considered normal from a clinical point of view. However, blood glucose alone is not sensitive enough and should be complemented by other parameters in future studies, including insulin activity and acetylcarnitine response.

### Regulation of gene expression

It is a fact that the cats from our colony are closely related to each other, leading to reduced genetic variability compared to cat cohorts from rotations in veterinary clinics. Therefore, even minor differences in gene expression regulation, as observed in the present study within the same animal between two time points, become apparent. The notably lower mapping rate in adipose tissue is likely attributable to the lower RNA concentrations typically obtained from adipose samples, on average only one third of that extracted from muscle tissue. In addition, minor contamination with muscle tissue during the biopsy process is plausible, given the anatomical proximity and the differences in cellular structure (i.e., large adipocytes versus myocytes). These factors may have contributed to the reduced mapping efficiency observed in adipose tissue. In adipose tissue, the only differentially expressed gene directly related to lipid metabolism was *monoacylglycerol O-acyltransferase 3*. This gene, which is important for fat absorption in the intestine, is downregulated in the obese cats compared to the lean cats, which would be a useful mechanism to avoid too high fat uptake. This implies a physiological reaction of the body to prevent further weight gain. In muscle tissue, significantly more genes were differentially expressed that are directly related to lipid metabolism, such as *fatty acid desaturase 3*, *LDL receptor related protein 5*, *perlipin 3*, *Ras related glycolysis inhibitor and calcium channel regulator*, *myostatin* and *bone morphogenetic protein 5*. Genes indirectly related to lipid metabolism were also affected, such as *galectin 1*, *SMAD family member 3*, *Notch* *receptor 3*, *natriuretic peptide receptor 3* and *selenium binding protein 1*, but again, these genes were mainly upregulated in the lean cats after ad libitum feeding, compared to the lean cats before ad libitum feeding, although there was no change in their BCS and body fat content. The differentially expressed genes in the obese cats are not directly related to lipid metabolism. It suggests that the absence of relevant differences in transcriptional regulation in target tissues observed between phenotypes over time are not a result of low measurement resolution but point indeed towards transcription playing only a minor role in the regulation of body fat in adult cats under the present experimental conditions. The most pronounced differences in gene expression in our cats were observed in comparison between muscle and adipose tissues, which is not surprising given the differences in function and metabolism of both tissues. Vink et al. [40] examined differences in gene expression in white adipose tissue before and after weight loss in fifty three overweight (body mass index 28–30) and obese (body mass index 31–35) men and women and found that gene sets related to mitochondrial function, adipogenesis, and immunity/inflammation were upregulated. Also in pigs, selected on the base of physical obesity traits among low and high extreme animals, Mentzel et al. [41] found a total of six miRNAs that were differentially expressed in subcutaneous adipose tissue between the lean and obese group of pigs. In contrast to these studies, our cats showed no clear effect on gene expression response as affected by changes of their body condition. This could be due to the fact that they were only slightly overweight compared to animals with extreme obesity, like in the study of Mentzel et al. [41]. The rationale behind performing differential expression analyses both between phenotypes (lean vs. obese) and within individuals (T1 vs. T2) is to separate a possible genetic predisposition from environmental changes in gene expression. By comparing lean and obese cats, the aim is to identify gene expression differences that may be due to genetic factors associated with the obesity phenotype. However, such differences could also be due to the simple amount of adipose tissue present. To account for this, we additionally analyzed samples from the same cat at two time points (T1 and T2), one when lean and one when obese. To exclude environmental influences, samples were also taken from phenotypically lean cats at two time points, even though they were lean at both T1 and T2. This dual approach allows us to better distinguish between expression patterns that are phenotype-driven and those that are dynamically regulated within an individual over time.

## Limitations of the study

The fact that the animals showed only a mild form of obesity during tissue collection may be a limiting factor. Furthermore, only a small number of individuals were investigated, but these were closely related to each other thereby reducing the genetic diversity thus allowing for higher resolution of gene expression measurements due to reduced background noise. Nevertheless, the small number of animals limits the extrapolation of the results of this study onto the entire cat population and therefore should be reproduced with a larger group of animals.

In retrospect, muscle tissue and white adipose tissue may have not been the most suitable targets to detect transcriptional differences between obesity phenotypes of adult cats. The notion to sample these particular tissues was guided by earlier studies in dogs and pigs, demonstrating varying gene expression in skeletal muscle and adipose tissue when comparing obese with lean individuals [41,42]. However, the present study is one of the first studies investigating transcriptomics in cats and in special of overweight cats of a cat family with segregating obese phenotype. If reproducible, our findings may shift the focus on transcriptomics research in obesity to other target tissues such as liver as the centre of metabolism. Liver was not sampled in our study since this biopsy is rather invasive and therefore more critical from an ethic’s perspective. Finally, future studies should pay more attention to potential post transcriptional regulation of cat obesity.

## Conclusion

In the present study no clear difference in the muscle and adipose tissue transcriptome of lean and obese cats was observed, except for time effects within respective individuals and tissues as well as the obvious differences in genetic regulation between two different types of tissue. Our observations in context to the earlier study by Opsomer et al. [34] on the same cat colony, which demonstrated positive correlation between maternal phenotype and kitten weight development, suggests that regulation of body fat in adult cats occurs at the post-transcriptional level thereby potentially involving an epigenetic component. Follow-up studies should investigate this idea. In addition, adult individuals from other mammal species should be investigated to understand whether this is specific for cats or a general phenomenon. Finally, investigations should be extended to more pronounced phenotypes of obesity.

## Supporting information

S1 Table Differentially expressed genes in muscle tissue in n = 6 cats (3 cats phenotype lean (GL), 3 cats phenotype obese (GO)). Timepoint 1 = GL and GO lean. Timepoint 2 = GL lean and GO obese. Annotation was performed with ensembl (https://www.ensembl.org).
(PDF)

S2 Table Differentially expressed genes in white adipose tissue in n = 6 cats (3 cats phenotype lean (GL), 3 cats phenotype obese (GO)). Timepoint 1 = GL and GO lean. Timepoint 2 = GL lean and GO obese. Annotation was performed with ensembl (https://www.ensembl.org).
(PDF)

S1 Fig Gene Set Enrichment Analysis of Biological Process (BP) terms in adipose tissue in n = 3 cats phenotype lean, comparing individuals at timepoint 1 versus timepoint 2. Ridgeplots illustrate the distribution of enriched gene sets across the ranked gene list, with genes more highly expressed at T1 on the left and at T2 on the right. Color denotes adjusted p-values (red = more significant).
(TIFF)

S2 Fig Gene Set Enrichment Analysis of Biological Process (BP) terms in adipose tissue in n = 6 cats (3 cats phenotype lean (GL), 3 cats phenotype obese (GO) comparing GO versus GL at timepoint 1. Ridgeplots illustrate the distribution of enriched gene sets across the ranked gene list, with genes more highly expressed for GO on the left and for GL on the right. Color denotes adjusted p-values (red = more significant).
(TIFF)

S3 Fig Gene Set Enrichment Analysis of Biological Process (BP) terms in adipose tissue in n = 3 cats phenotype obese, comparing individuals at timepoint 1 versus timepoint 2. Ridgeplots illustrate the distribution of enriched gene sets across the ranked gene list, with genes more highly expressed at T1 on the left and at T2 on the right. Color denotes adjusted p-values (red = more significant).
(TIFF)

S4 Fig Gene Set Enrichment Analysis of Biological Process (BP) terms in adipose tissue in n = 6 cats (3 cats phenotype lean (GL), 3 cats phenotype obese (GO) comparing GO versus GL at timepoint 2. Ridgeplots illustrate the distribution of enriched gene sets across the ranked gene list, with genes more highly expressed for GO on the left and for GL on the right. Color denotes adjusted p-values (red = more significant).
(TIFF)

S5 Fig Gene Set Enrichment Analysis of Biological Process (BP) terms in muscle tissue in n = 3 cats phenotype lean, comparing individuals at timepoint 2 versus timepoint 1. Ridgeplots illustrate the distribution of enriched gene sets across the ranked gene list, with genes more highly expressed at T2 on the left and at T1 on the right. Color denotes adjusted p-values (red = more significant).
(TIFF)

S6 Fig Gene Set Enrichment Analysis of Biological Process (BP) terms in muscle tissue in n = 6 cats (3 cats phenotype lean (GL), 3 cats phenotype obese (GO) comparing GO versus GL at timepoint 1. Ridgeplots illustrate the distribution of enriched gene sets across the ranked gene list, with genes more highly expressed for GO on the left and for GL on the right. Color denotes adjusted p-values (red = more significant).
(TIFF)

S7 Fig Gene Set Enrichment Analysis of Biological Process (BP) terms in muscle tissue in n = 3 cats phenotype obese, comparing individuals at timepoint 1 versus timepoint 2. Ridgeplots illustrate the distribution of enriched gene sets across the ranked gene list, with genes more highly expressed at T1 on the left and at T2 on the right. Color denotes adjusted p-values (red = more significant).
(TIFF)

S8 Fig Gene Set Enrichment Analysis of Biological Process (BP) terms in muscle tissue in n = 6 cats (3 cats phenotype lean (GL), 3 cats phenotype obese (GO) comparing GO versus GL at timepoint 2. Ridgeplots illustrate the distribution of enriched gene sets across the ranked gene list, with genes more highly expressed for GO on the left and for GL on the right. Color denotes adjusted p-values (red = more significant).
(TIFF)
