# Supplementary material for: Transcriptional regulation in skeletal muscle and adipose tissue of lean and obese colony cats
Source: PLoS One. 2026 Mar 27;21(3):e0331028. doi: 10.1371/journal.pone.0331028 (PMC13028413; doi:10.1371/journal.pone.0331028)
Supplement: S1 Table — Timepoint 1 = GL and GO lean. Timepoint 2 = GL lean and GO obese. Annotation was performed with ensembl (https://www.ensembl.org). (PDF) [file pone.0331028.s001.pdf]

Muscle tissue	Upregulated	Downregulated
**GL T2 compared to GL T1**	basal cell adhesion molecule (ENSG00000187244)<br><br>protein phosphatase, Mg2+/ Mn2+ dependent 1K (ENSG00000163644)<br><br>mitoguardin 2 (ENSG00000148343)<br><br>lamin A/C (ENSG00000160789)<br><br>mitogen-activated protein kinase 8 interacting protein 3 (ENSG00000138834)<br><br>dehydrogenase/reductase 3 (ENSG00000162496)<br><br>BCL9 like (ENSG00000186174)<br><br>methyl-CpG binding domain protein 6 (ENSG00000166987)<br><br>synaptojanin 2 (ENSG00000078269)<br><br>galectin 1 (ENSG00000100097)<br><br>capicua transcriptional repressor (ENSG00000079432)<br><br>SMAD family member 3 (ENSG00000166949)<br><br>LDL receptor related protein 5 (ENSG00000162337)<br><br>fatty acid desaturase 3 (ENSG00000221968)<br><br>notch receptor 3 (ENSG00000074181)<br><br>2'-5'-oligoadenylate synthetase 3 (ENSG00000111331)<br><br>ST6 N-acetylgalactosaminide alpha-2,6-sialyltransferase 4 (ENSG00000136840)	frizzled related protein (ENSG00000162998)<br><br>gastrokine 1 (ENSG00000169605)<br><br>collagen alpha-2(XI) chain-like (ENSG00000204248)<br><br>Rho GTPase activating protein 28 (ENSG00000088756)<br><br>myostatin (ENSG00000138379)<br><br>bone morphogenetic protein 5 (ENSG00000112175)

	heat shock protein family B (small) member 6 (ENSG00000004776)<br><br>smoothelin like 1 (ENSG00000214872)<br><br>perilipin 3 (ENSG00000105355)<br><br>growth regulating estrogen receptor binding 1 (ENSG00000196208)<br><br>platelet basic protein (ENSG00000163736)<br><br>collagen like tail subunit of asymmetric acetylcholinesterase (ENSG00000206561)<br><br>zinc finger protein 385A (ENSG00000161642)<br><br>integrin subunit alpha 2b (ENSG00000005961)<br><br>DM1 protein kinase (ENSG00000104936)<br><br>cadherin 16 (ENSG00000166589)<br><br>selenium binding protein 1 (ENSG00000143416)<br><br>RRAD, Ras related glycolysis inhibitor and calcium channel regulator (ENSG00000166592)	
**GO T1 over GO T2**	Podoplanin (ENSG00000162493)<br><br>tenomodulin (ENSG00000000005)<br><br>fibromodulin (ENSG00000122176)<br><br>thrombospondin 4 (ENSG00000113296)	ALG13 UDP-N-acetylglucosaminyltransferase subunit (ENSG00000101901)<br><br>dual oxidase 2 (ENSG00000140279)
**GO T1 over GL T1**	potassium voltage-gated channel subfamily Q member 1 (ENSG00000053918)<br><br>TAP binding protein (ENSG00000231925)	tetraspanin 19 (ENSG00000231738)<br><br>tapasin (ENSG00000231925)<br><br>calcium voltage-gated channel auxiliary subunit beta 4 (ENSG00000182389)
**GO T2 over GL T2**	frizzled related protein (ENSG00000162998)	

	natriuretic peptide receptor 3 (ENSG00000113389)	
