# Supplementary material for: Transcriptional regulation in skeletal muscle and adipose tissue of lean and obese colony cats
Source: PLoS One. 2026 Mar 27;21(3):e0331028. doi: 10.1371/journal.pone.0331028 (PMC13028413; doi:10.1371/journal.pone.0331028)
Supplement: S2 Table — Timepoint 1 = GL and GO lean. Timepoint 2 = GL lean and GO obese. Annotation was performed with ensembl (https://www.ensembl.org). (PDF) [file pone.0331028.s002.pdf]

Adipose tissue	Upregulated	Downregulated
**GL T1 over GL T2**		zinc transporter SLC39A7-like (ENSG00000227402)
**GO T1 over GO T2**		
**GO T1 over GL T1**	sushi domain containing 2 (ENSG00000099994)<br><br>interferon alpha inducible protein 6 (ENSG00000126709)<br><br>zinc transporter SLC39A7-like (ENSG00000227402)<br><br>bone marrow stromal cell antigen 2 (ENSG00000130303)	
**GO T2 over GL T2**		Tapasin (ENSG00000231925)<br><br>monoacylglycerol O-acyltransferase 3 (ENSG00000106384)
