# Supplementary figures and images for: Transcriptional regulation in skeletal muscle and adipose tissue of lean and obese colony cats
Source: PLoS One. 2026 Mar 27;21(3):e0331028. doi: 10.1371/journal.pone.0331028 (PMC13028413; doi:10.1371/journal.pone.0331028)

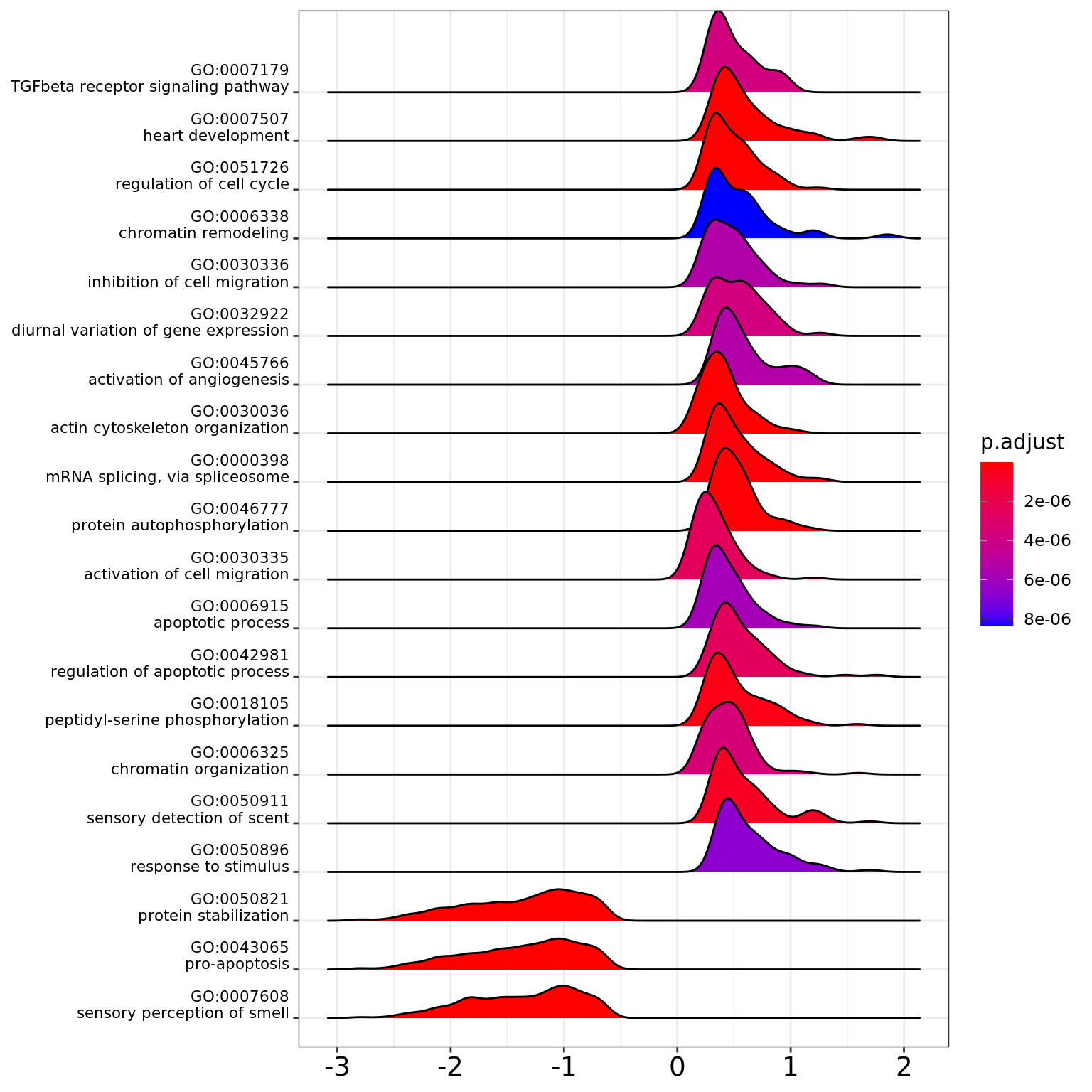

Supplement: S1 Fig — Ridgeplots illustrate the distribution of enriched gene sets across the ranked gene list, with genes more highly expressed at T1 on the left and at T2 on the right. Color denotes adjusted p-values (red = more significant). (TIFF) [file pone.0331028.s003.tiff]

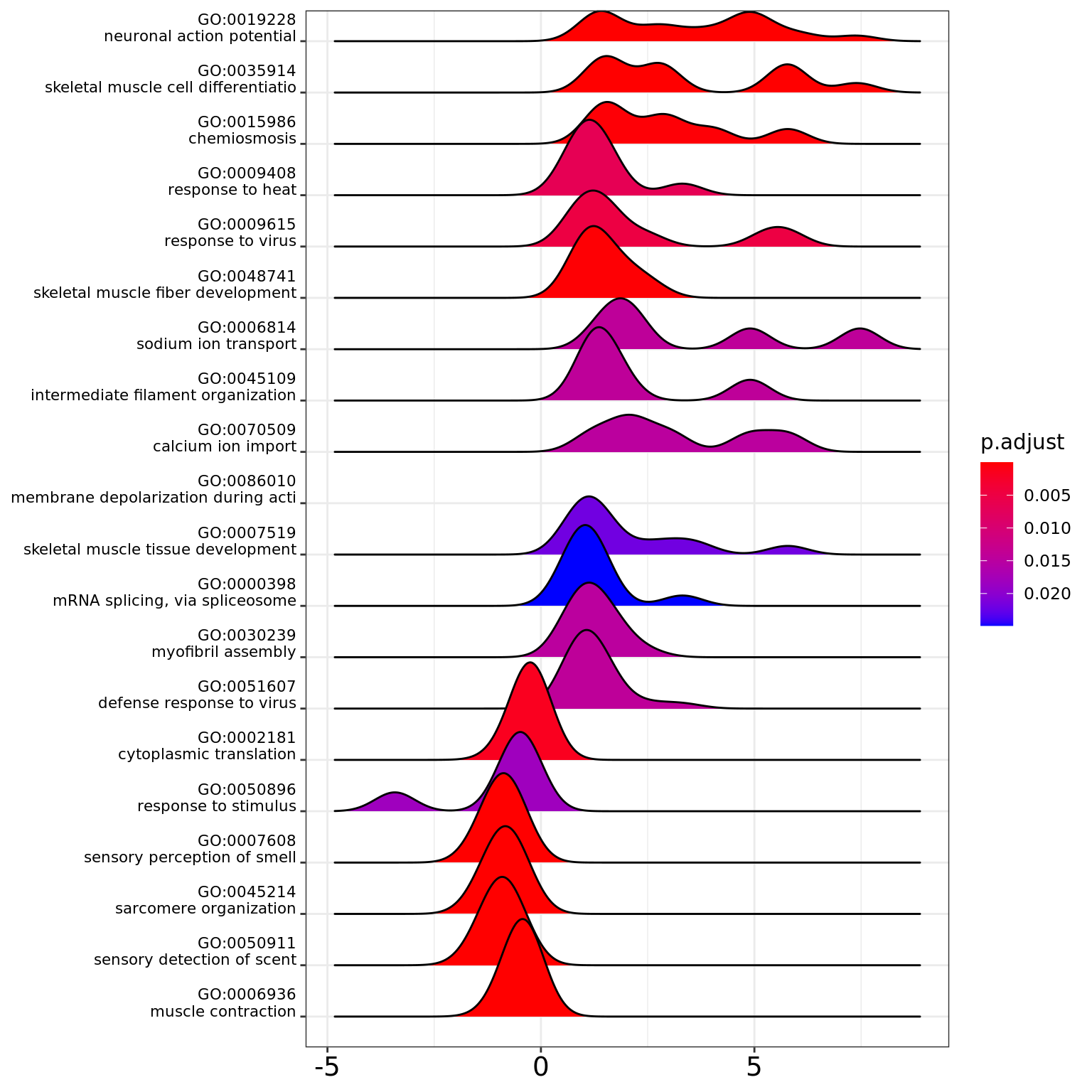

Supplement: S2 Fig — Ridgeplots illustrate the distribution of enriched gene sets across the ranked gene list, with genes more highly expressed for GO on the left and for GL on the right. Color denotes adjusted p-values (red = more significant). (TIFF) [file pone.0331028.s004.tiff]

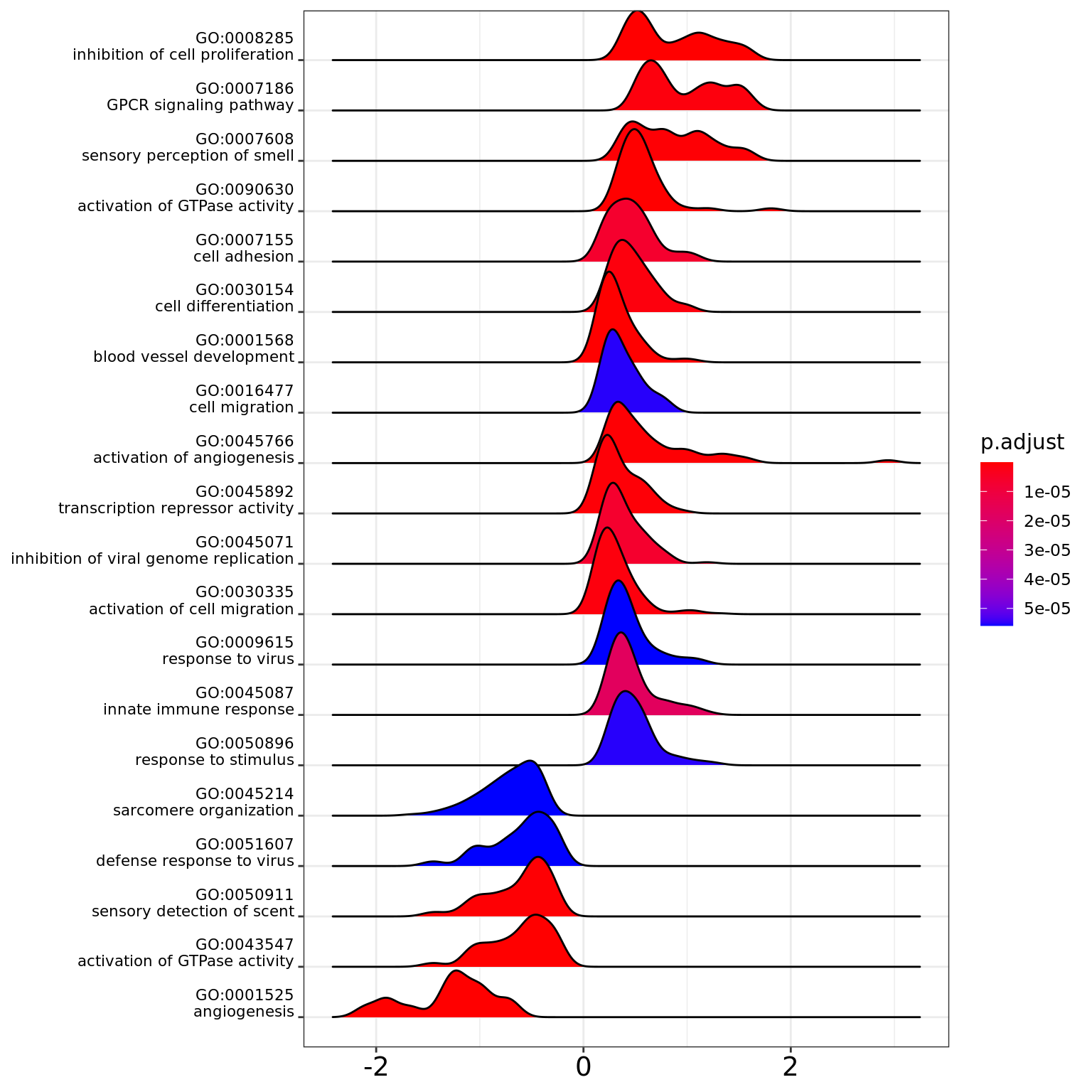

Supplement: S3 Fig — Ridgeplots illustrate the distribution of enriched gene sets across the ranked gene list, with genes more highly expressed at T1 on the left and at T2 on the right. Color denotes adjusted p-values (red = more significant). (TIFF) [file pone.0331028.s005.tiff]

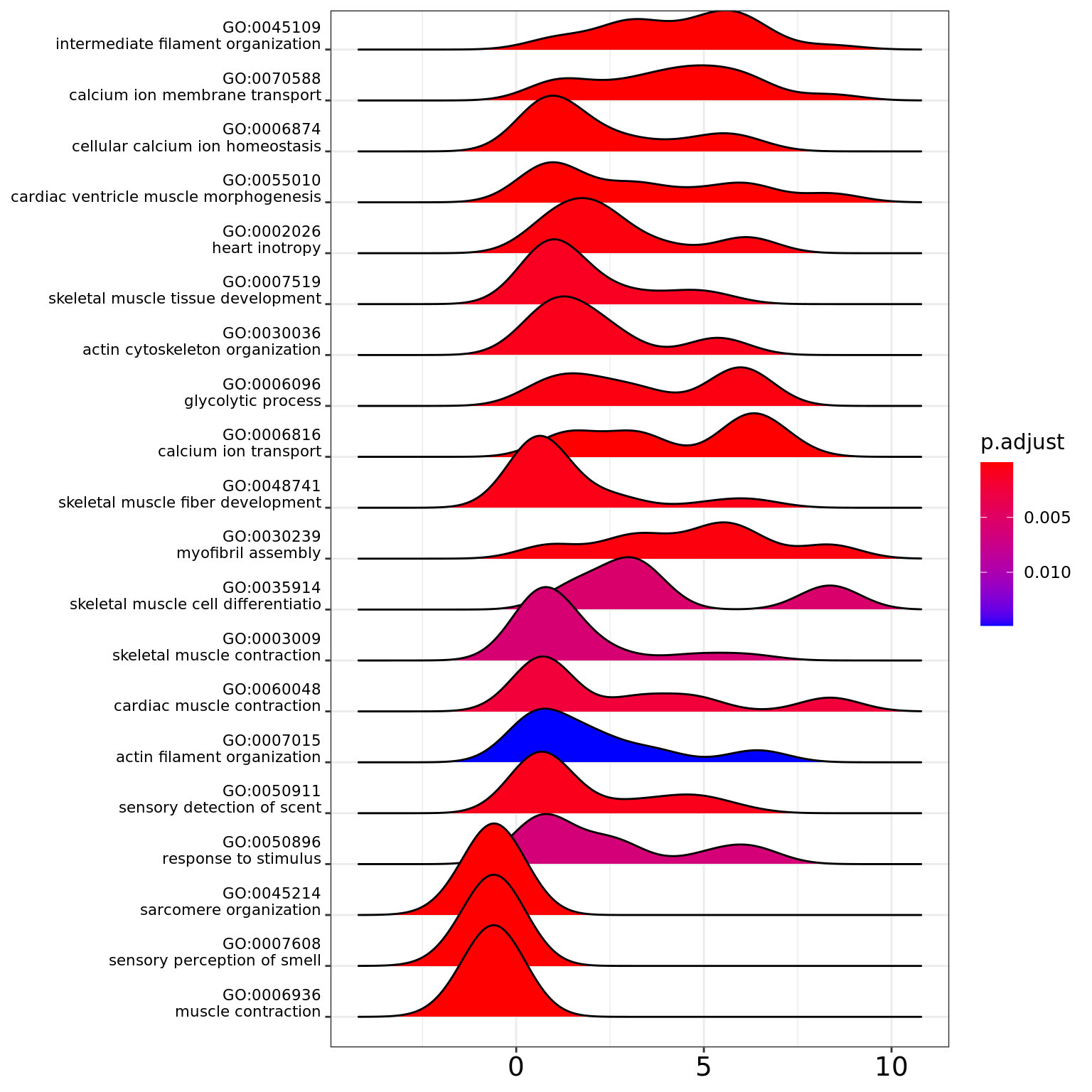

Supplement: S4 Fig — Ridgeplots illustrate the distribution of enriched gene sets across the ranked gene list, with genes more highly expressed for GO on the left and for GL on the right. Color denotes adjusted p-values (red = more significant). (TIFF) [file pone.0331028.s006.tiff]

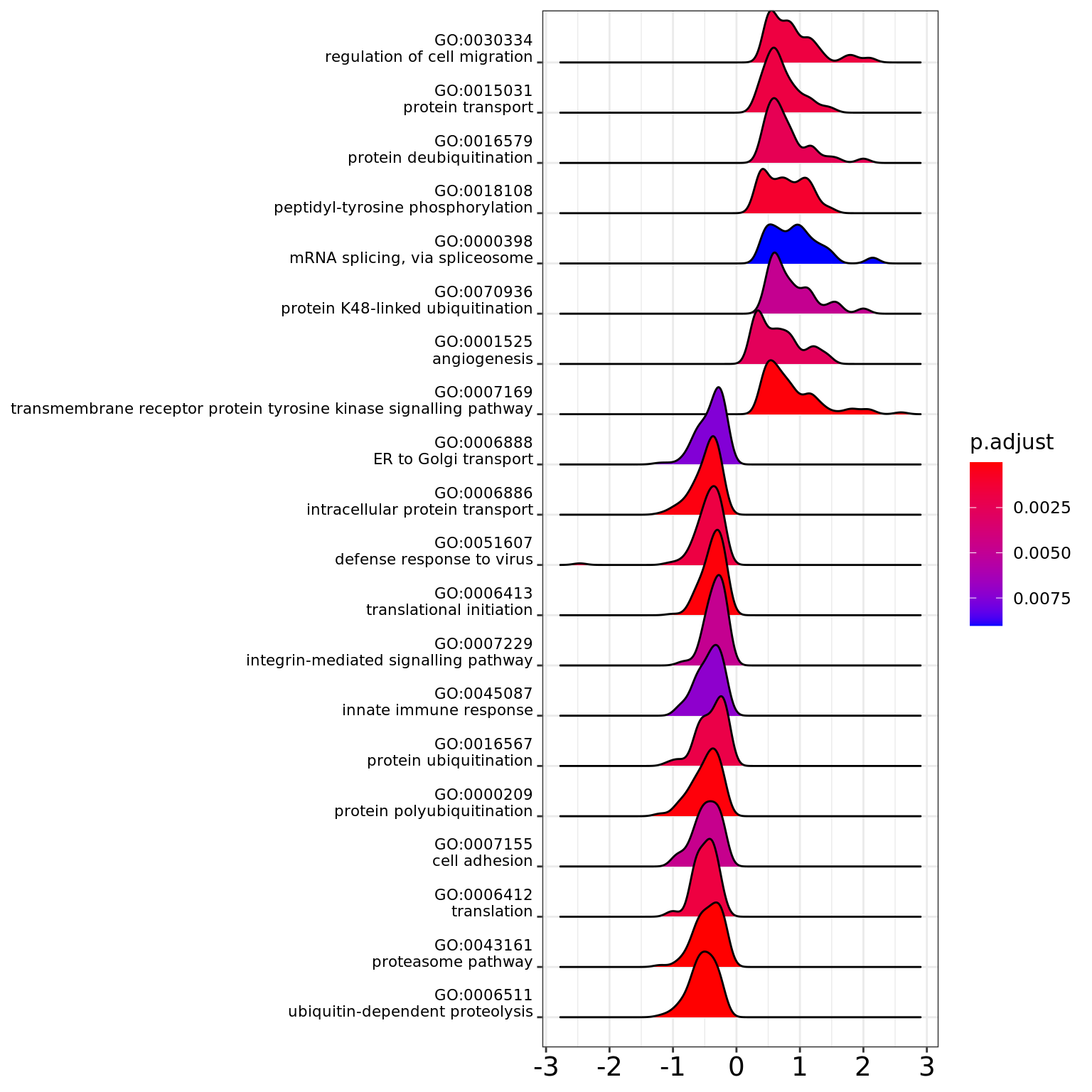

Supplement: S5 Fig — Ridgeplots illustrate the distribution of enriched gene sets across the ranked gene list, with genes more highly expressed at T2 on the left and at T1 on the right. Color denotes adjusted p-values (red = more significant). (TIFF) [file pone.0331028.s007.tiff]

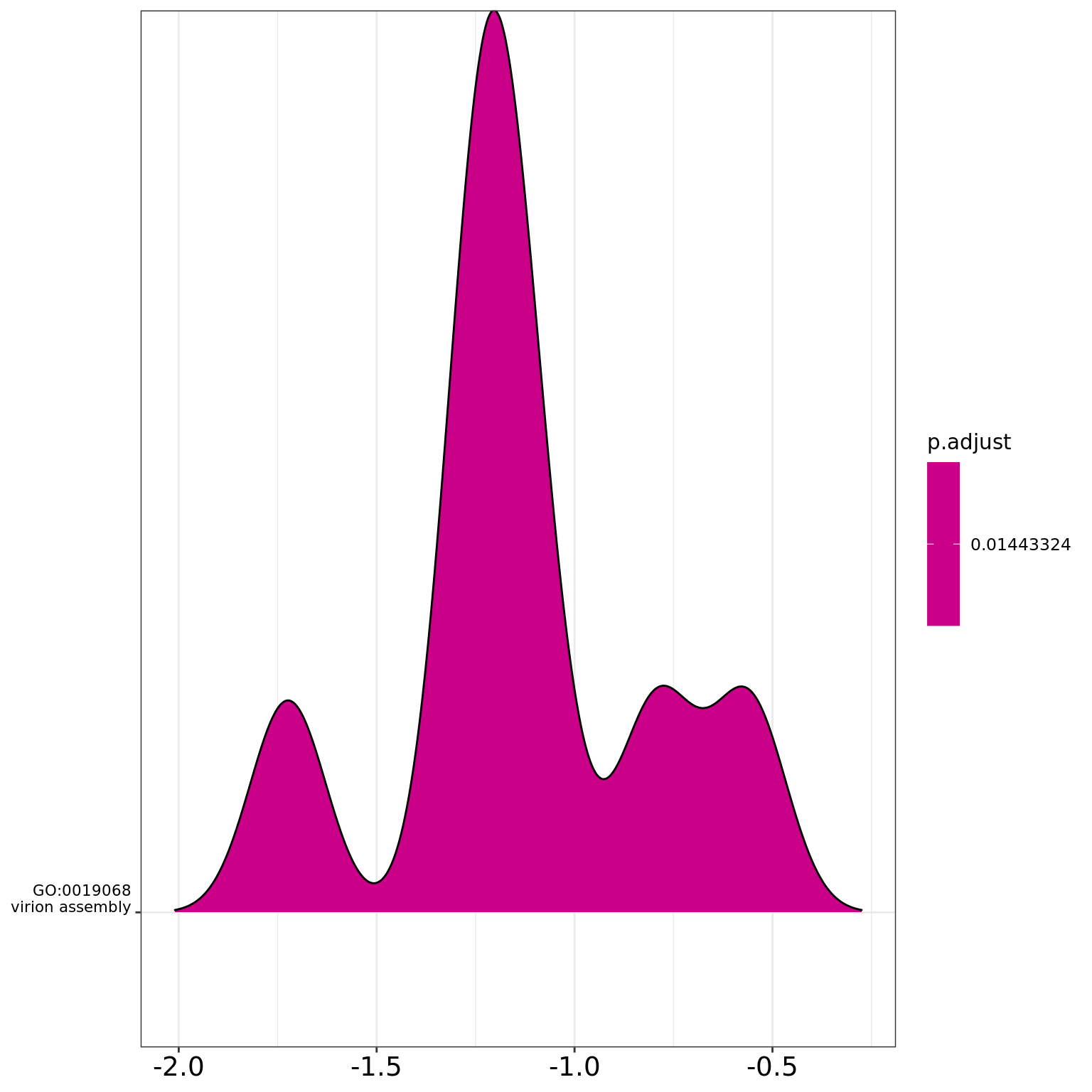

Supplement: S6 Fig — Ridgeplots illustrate the distribution of enriched gene sets across the ranked gene list, with genes more highly expressed for GO on the left and for GL on the right. Color denotes adjusted p-values (red = more significant). (TIFF) [file pone.0331028.s008.tiff]

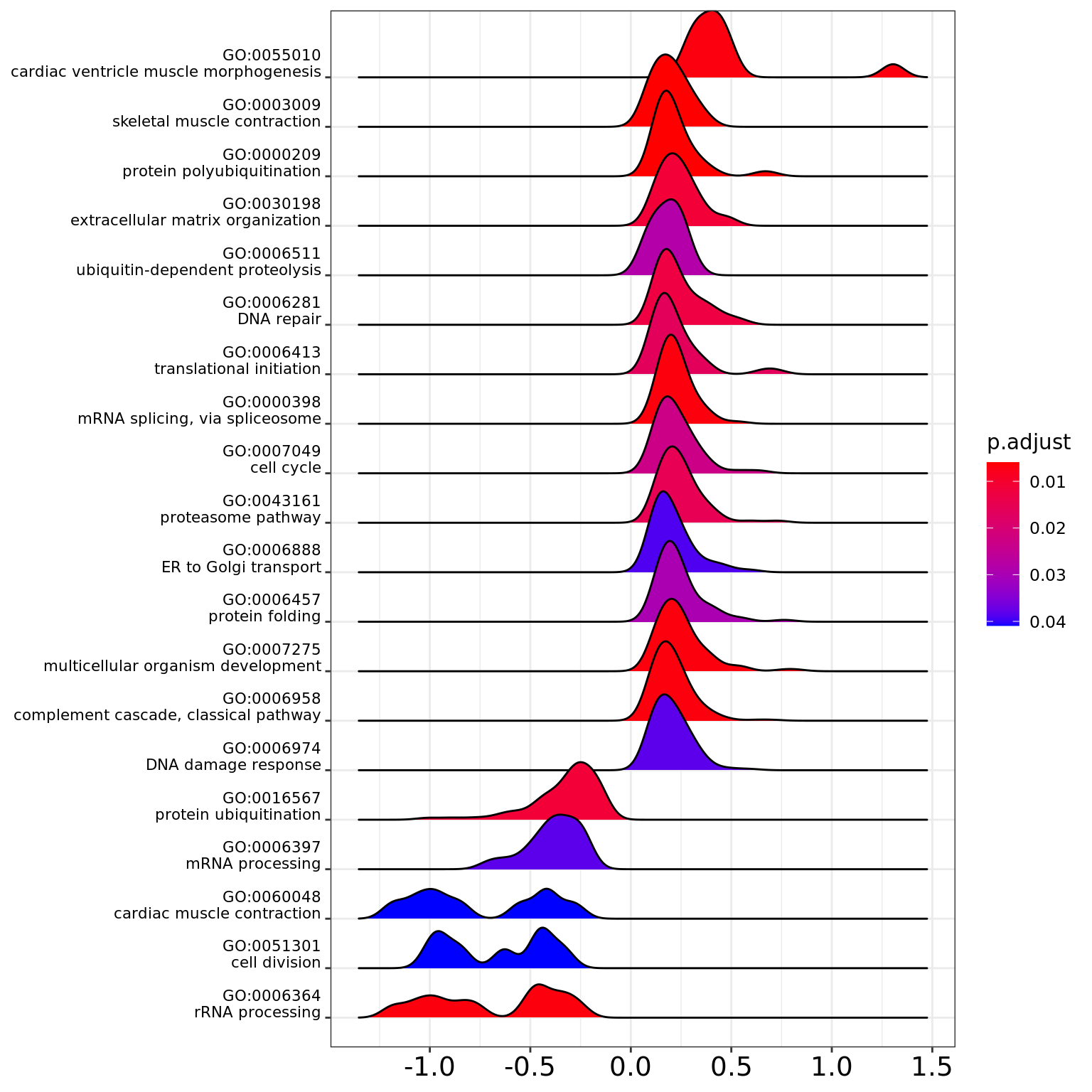

Supplement: S7 Fig — Ridgeplots illustrate the distribution of enriched gene sets across the ranked gene list, with genes more highly expressed at T1 on the left and at T2 on the right. Color denotes adjusted p-values (red = more significant). (TIFF) [file pone.0331028.s009.tiff]

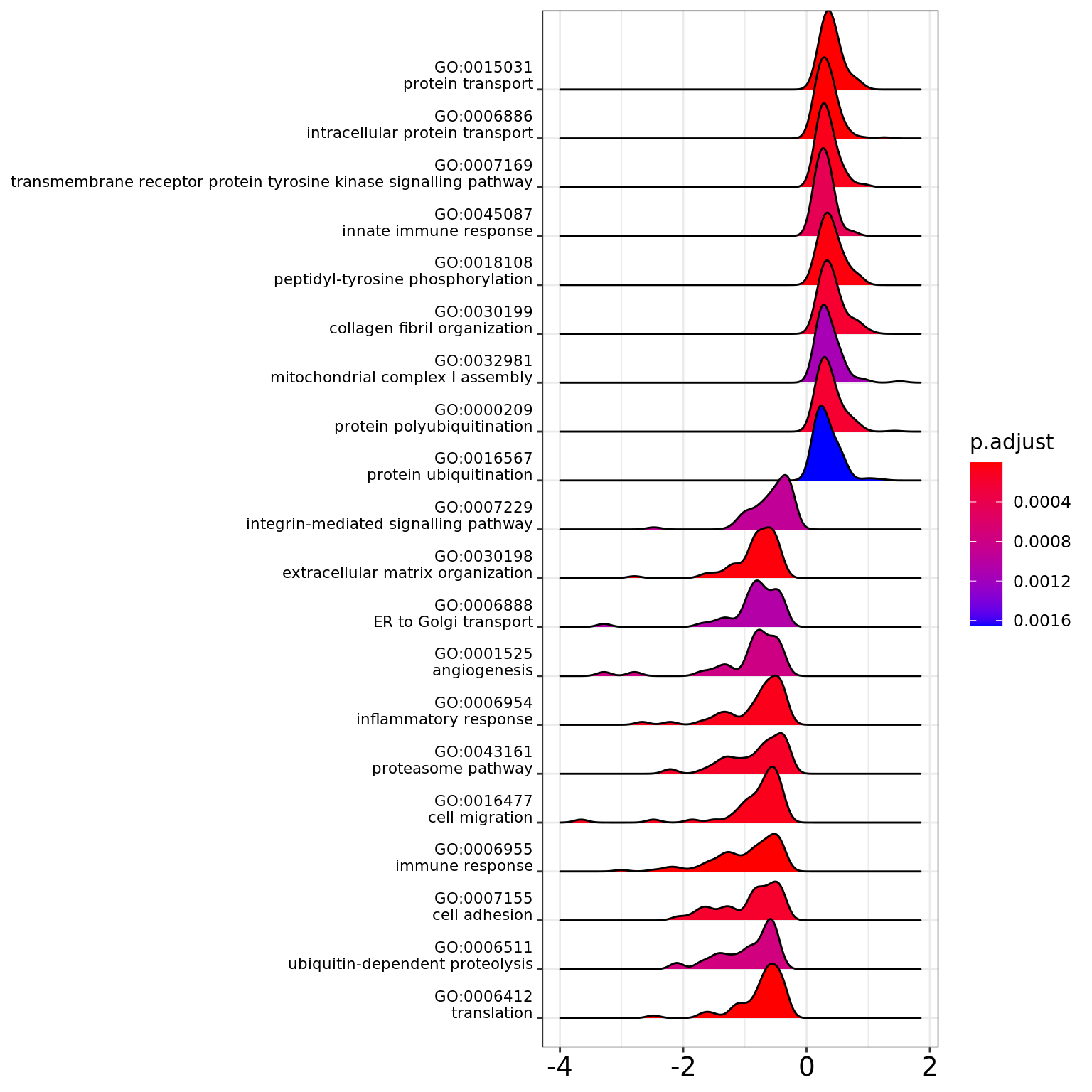

Supplement: S8 Fig — Ridgeplots illustrate the distribution of enriched gene sets across the ranked gene list, with genes more highly expressed for GO on the left and for GL on the right. Color denotes adjusted p-values (red = more significant). (TIFF) [file pone.0331028.s010.tiff]
